# Patient safety and quality care: Time to focus on nonventilator hospital-acquired pneumonia

**DOI:** 10.1017/ash.2022.169

**Published:** 2022-05-16

**Authors:** Karen Giuliano, Dian Baker

## Abstract

**Background:** A growing body of evidence has reported on the harm and cost of nonventilator hospital-acquired pneumonia (NVHAP), currently the most common hospital-acquired infection (HAI). Although the US Congress and the Center for Medicare and Medicaid Services (CMS) have acted to reduce rates of some HAIs through the Hospital-Acquired Condition Reduction Program (HACRP), NVHAP is not currently included. Thus, most hospitals do not engage in active prevention. Here, we report the findings from our analysis of Medicare claims data on hospital length of stay (LOS), cost for patients with hospital-acquired pneumonia (HAP), including both ventilator-associated pneumonia and NVHAP, and mortality. **Methods:** We used Medicare claims data for Federal Fiscal Year 2019 for inpatient and postdischarge services. Beneficiaries who died, were without continuous Medicare Part A and B enrollment, and patients eligible for Medicare for end-stage renal disease were excluded. Inpatient payments and 30-, 60-, and 90-day postdischarge episodes for 2,457 beneficiaries with HAP were examined and compared to a non-HAP control group of 2,457 beneficiaries. Groups were matched on age, sex, race, and the diagnosis-related group (DRG) for their index hospitalization. **Results:** Most HAP was NVHAP (N = 2,222; 89%) versus VAP (N = 275; 11%). LOS stay was significantly (p HAP patients were 2.8 times more likely to die vs non-HAP. **Conclusions:** These findings provide additional support to previous research on the harm and cost associated with NVHAP. Previous HACRP HAI initiatives, such as catheter-associated urinary tract infection (CAUTI) and surgical-site infection (SSI), have resulted in measurable HAI reductions. Although recent evidence-based NVHAP and initiatives indicate that NVAHP is largely preventable, to date, no acute-care inpatient hospital quality improvement program implemented by Medicare includes measures for NVHAP prevention. The time is right to include NVHAP as an HACRP HAI initiative.

**Funding:** Stryker

**Disclosures:** None

